# Antimicrobial activity of *Limosilactobacillus fermentum* strains isolated from the human oral cavity against* Streptococcus mutans*

**DOI:** 10.1038/s41598-023-35168-7

**Published:** 2023-05-17

**Authors:** Do-Young Park, Jiyoung Hwang, Yunji Kim, Dahye Lee, Young-Youn Kim, Hye-Sung Kim, Inseong Hwang

**Affiliations:** 1 DOCSmedi Co., Ltd., Goyang-Si, South Korea; 2 Apple Tree Institute of Biomedical Science, Apple Tree Medical Foundation, Goyang-Si, South Korea; 3 Apple Tree Dental Hospital, Apple Tree Medical Foundation, Goyang-Si, South Korea

**Keywords:** Microbiology, Dentistry

## Abstract

Oral probiotics have been recently gaining much attention owing to their potential to inhibit the progression of dental caries by controlling the cariogenic effects of *Streptococcus mutans*. We isolated and genotypically identified 77 lactic acid bacteria including 12 *Limosilactobacillus fermentum* probiotic candidates from the oral cavity of healthy volunteers. Among the 12 *L. fermentum* isolates, nine isolates effectively inhibited the growth of *S. mutans* via hydrogen peroxide (H_2_O_2_) production. The others neither suppressed the growth of *S. mutans* nor produced H_2_O_2_. Eight out of the nine H_2_O_2_-producing *L. fermentum* isolates exhibited strong adherence to oral epithelial KB cells while inhibiting the adherence of *S. mutans* to KB cells. The eight H_2_O_2_-producing isolates were neither haemolytic based on a blood-agar test, cytotoxic according to lactate dehydrogenase assay, nor resistant to eight antibiotics represented by the European Food Safety Authority guideline, indicating that the isolates have potential to suppress the cariogenesis driven by *S. mutans* while providing general probiotic benefits.

## Introduction

The oral cavity, a gateway for microorganisms to the human internal system, provides a suitable environment including, but not limited to, humidity, temperature, pH, and nutrients for the cultivation of commensal microbes^1^. As such, the human host and its microbiome have established a unique ecology as a combined unit with a bidirectional relationship^2,3^. Studies have found that microbial dysbiosis, an ecological imbalance between pathogenic and beneficial microorganisms, can lead to oral diseases, such as caries and periodontal disease^3^. On the other hand, many antibiotics, routinely used in dentistry to prevent bacteraemia, can increase the number of antibiotic-resistant strains while breaking the healthy ecology of the oral cavity, leading to systemic infections^4,5^.

Dental caries is one of the highly prevalent non-communicable and chronic diseases, demanding expensive healthcare budgets in industrialized countries^6,7^. As of 2019, untreated dental caries continues to be a significant global public health issue with more than 3 billion new cases (48% increase), 2 billion prevalent cases (46% increase), and 2 million years lived with disabilities (YLDs) (46% increase) since 1990^8^. Dental caries, often known as tooth decay or cavities, is determined by the imbalanced demineralization caused by pathogenic factors including acidogenic and aciduric bacteria such as mutans streptococci (MS)^9^.

*Streptococcus mutans*, a facultative anaerobic Gram-positive bacterium found in the human oral cavity, belongs to the MS group, has an increased ability to adhere to the tooth surface, metabolizes sucrose to produce lactic acid that weakens tooth enamel, and converts sucrose to a sticky insoluble dextran-based polysaccharide that allows plaque formation^10,11^. Thus, *S. mutans* is considered to be a primary agent that initiates and develops dental caries^12^. Of note, numerous investigations have demonstrated that dental caries is caused by microbial dysbiosis in the oral cavity^13^. The ecological imbalance, a temporary status when cariogenic pathobionts outnumber commensal symbionts, can transform non-cariogenic plaque into a cariogenic one. Thus, it is important to contain the spreading of cariogenic bacteria such as MS while keeping commensal bacteria from developing into pathobionts. Consequently, much effort has been focused on inhibiting *S. mutans* growth using probiotics as a biological intervention that minimizes the disruption of ecological balance in the oral cavity^12,14–20^.

The genus *Limosilactobacillus*, a member of the family *Lactobacillaceae*, is a thermophilic and heterofermentative genus of the lactic acid bacteria (LAB) and comprises 31 species, some of which are relevant to industry and medicine^21^. *L. fermentum* is one of the most widely commercialized heterofermentative limocilactobacilli, commonly isolated from various human body locations and fermenting materials^22^. The bacterium produces diverse antimicrobial peptides, bacteriocins, and, in some cases, H_2_O_2_ upon exposure to molecular oxygen^23^. While many species of LAB can generate enough H_2_O_2_ to inhibit the growth of *S. mutans*^16,17,20,24^, studies on the oral probiotic function of *L. fermentum* via H_2_O_2_ against *S. mutans* are still lacking.

The Human Microbiome Project (HMP), supported by the National Institutes of Health (NIH) from 2007 to 2016, has examined a range of microbial habitats in the oral cavity, such as saliva, buccal mucosa, keratinized gingiva, palate, tonsils, throat and tongue soft tissues, and supra-and subgingival dental plaque^25^. The expanded HMP (HMP-II) further selected buccal mucosa, supragingival plaque, and tongue dorsum among the oral landscape for the analysis of body-wide strain diversity, showing that strain profiles were site-specific and stable over time^1,26,27^. The resulting microbial profiles in the oral cavity are available on the expanded Human Oral Microbiome Database (eHOMD v3, http://homd.org/, accessed on 10 December 2022), currently enlisting 774 species of which 58% are formally named, 16% unnamed but cultivated, and 26% known but uncultivated. The human tongue dorsum is rich in papillae on the non-keratinized epithelial layer that absorb small molecules including postbiotic compounds, enabling interaction with the host^1,28^. Thus, the role of tongue-coating (TC) microbiota has gained much traction because of its association with metabolic disorders^28^.

This study aimed to isolate new probiotic LAB with antimicrobial activity against *S. mutans* from human TC biospecimens obtained from 100 healthy volunteers. We successfully isolated 77 LAB of which species were identified by 16S rRNA partial sequencing and found three new species that are not listed in HOMD. We found that eight out of 12 *L. fermentum* isolates inhibited *S. mutans* growth by producing H_2_O_2_, dramatically reduced the formation of *S. mutans* plaque, and suppressed the adherence of *S. mutans* to oral epithelial cells while satisfying the safety guidelines on haemolysis, cytotoxicity, and antibiotics resistance recommended by the Food and Agriculture Organization of the United Nations (FAO)/World Health Organization (WHO) and the European Food Safety Authority (EFSA)^29^.

## Results

### Isolation of LAB species from the human tongue-coating biospecimens

**Figure 1:**
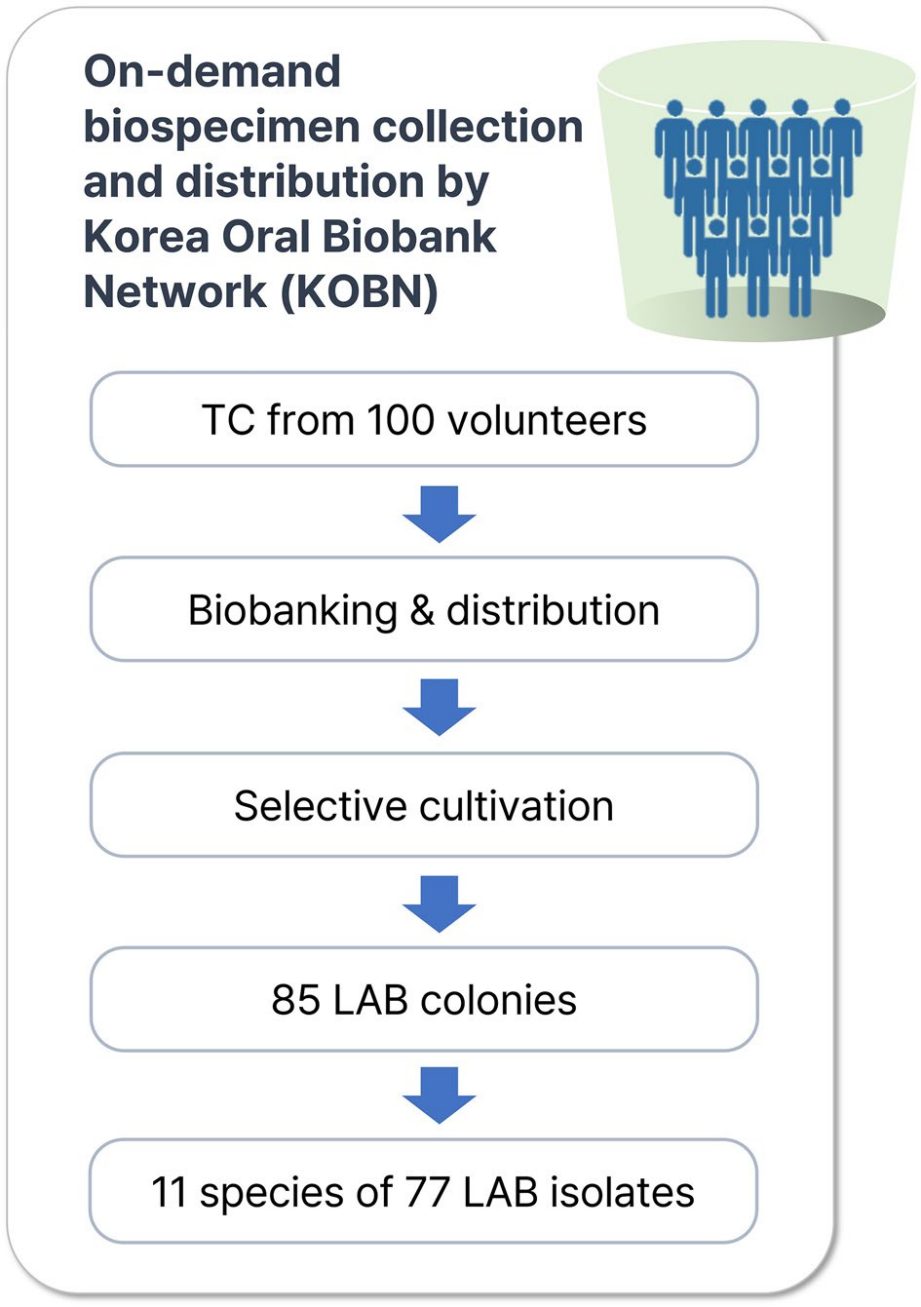
Isolation of LAB from human tongue coating biospecimens collected and distributed by Korea Oral Biobank Network (KOBN).

We initially isolated a total of 85 colonies from the human tongue coating specimens collected and distributed by the Korea Oral Biobank Network (KOBN) (Fig. 1). Successive cultivation resulted in 77 stable LAB isolates, which belong to eight different genera and 11 different species according to the reclassified nomenclature (Table 1) ^21^. *Lacticaseibacillus rhamnosus* (*n* = 17) showed the highest incidence, followed by *Lactobacillus gasseri* (*n* = 14), *Limosilactobacillus vaginalis* (*n* = 13), *L. fermentum* (*n* = 12), *Lactiplantibacillus plantarum* (*n* = 9), *Lacticaseibacillus paracasei* (*n* = 4), *Ligilactobacillus salivarius* (*n* = 3), *Limosilactobacillus mucosae* (*n* = 2), *Levilactobacillus brevis* (*n* = 1), *Lentilactobacillus sunkii* (*n* = 1), and *Liquorilactobacillus nagelii* (*n* = 1). Among them, *L. mucosae*, *L. sunkii*, and *L. nagelii* are newly found species not listed in HOMD.

**Table 1:** The list of LAB isolates identified by 16S rRNA sequencing analysis.

Species	No. of isolates	Listed in HOMD
*Lacticaseibacillus rhamnosus*	17	Yes
*Lactobacillus gasseri*	14	Yes
*Limosilactobacillus vaginalis*	13	Yes
*Limosilactobacillus fermentum*	12	Yes
*Lactiplantibacillus plantarum*	9	Yes
*Lacticaseibacillus paracasei*	4	Yes
*Ligilactobacillus salivarius*	3	Yes
*Limosilactobacillus mucosae*	2	No
*Levilactobacillus brevis*	1	Yes
*Lentilactobacillus sunkii*	1	No
*Liquorilactobacillus nagelii*	1	No
Total	77	

### Selection of LAB isolates inhibiting the growth of *S. mutans*

**Figure 2:**
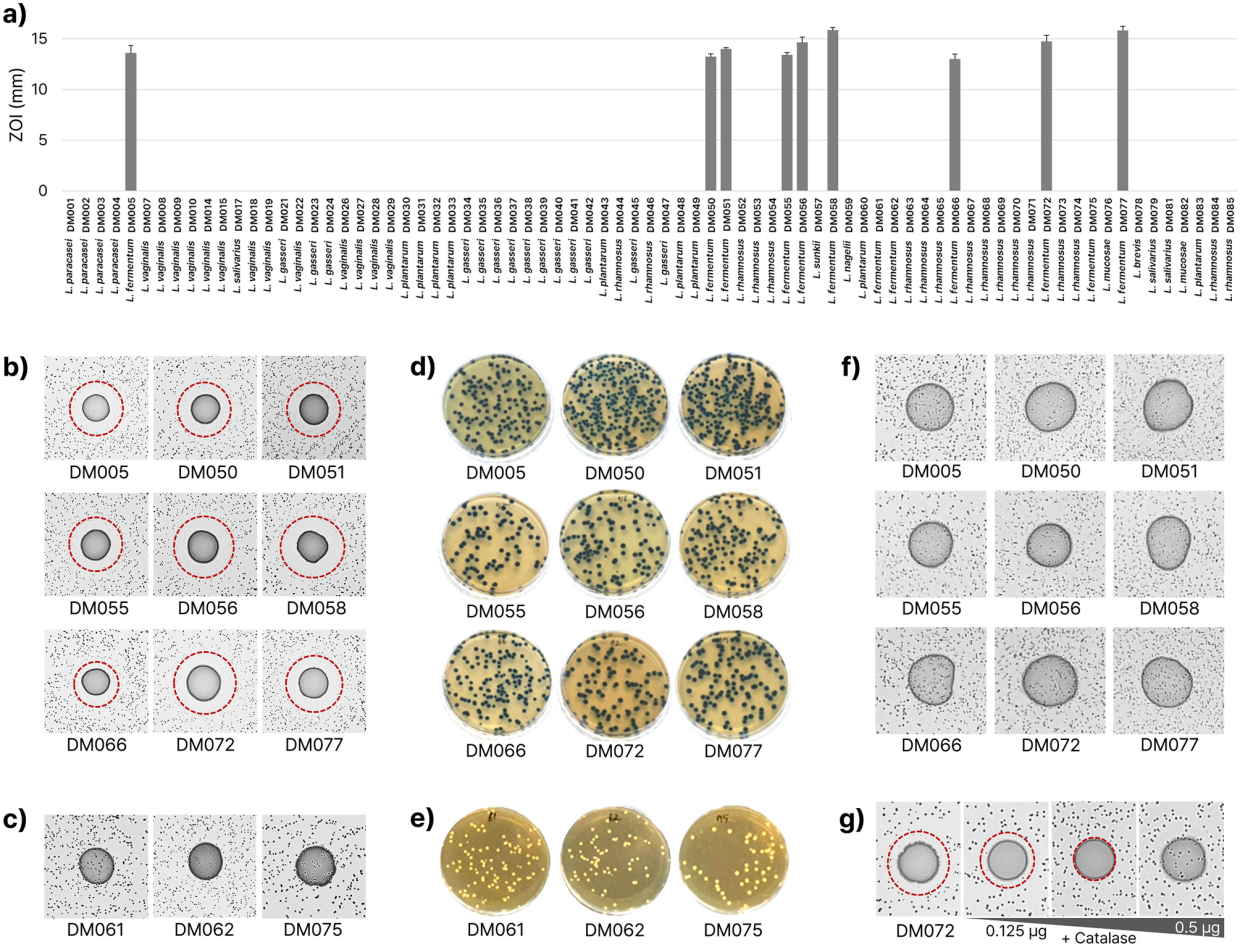
*L. fermentum*-mediated *S. mutans* growth inhibition is mediated by H_2_O_2_. (**a**) Initial antimicrobial functional screening of 85 isolates against *S. mutans* by measuring ZOI around the 3 μL of each culture droplet. The *S. mutans* ZOIs (indicated by red broken lines) appeared only in nine *L. fermentum* species (**b**) except DM061, DM062, and DM075 (**c**). The nine functional *L. fermentum* strains produced H_2_O_2_, yielding blue colours owing to TMB oxidation in the presence of peroxidase and 5% molecular oxygen (**d**) while the non-functional three isolates did not (**e**). (**f**) The inhibitory function of the nine *L. fermentum* strains disappeared in the presence of catalase, resulting in normal colonization of *S. mutans* within the spot areas. (**g**) The diameter of ZOI decreased as the concentration of catalase increased in the case of *L. fermentum* DM072, corroborating the inhibitory effect of H_2_O_2_ against *S. mutans* growth.

The antimicrobial activity of 77 LAB isolates against *S. mutans* was screened by the customized zone of inhibition (ZOI) test. Among the 12 *L. fermentum* isolates, nine *L. fermentum* isolates (DM005, DM050, DM051, DM055, DM056, DM058, DM066, DM072, and DM077) showed clear ZOIs (Fig. 2a,b). In contrast, the rest three (DM061, DM062, and DM075) failed to form visible ZOIs, allowing *S. mutans* to grow together with the isolates (Fig. 2c). These results implicated that the nine functional isolates, unlike the other inactive ones, produce certain antibacterial compounds against *S. mutans*. Interestingly, the three non-functional isolates survived at pH 2.5 while the nine functional isolates only survived at pH 3.0 than the three non-functional isolates (Supplementary Fig. S1), suggesting differences in proton pumping, proton consumption, or alkali production pathways between the two groups^30^.

### Inhibition of *S. mutans* growth by H_2_O_2_-producing *L. fermentum* isolates

Several studies previously demonstrated that the growth of *S. mutans* was inhibited by H_2_O_2_-producing bacteria^20,31^. To evaluate whether H_2_O_2_ production is linked to antibacterial activity against *S. mutans,* we used de Man-Rogosa-Sharpe (MRS) agar supplemented with 3,3’,5,5’-tetramethylbenzidine (TMB) to colourise the H_2_O_2_-producing colonies. Upon exposure to molecular oxygen, the colonies of the nine *L. fermentum* isolates turned into blue colours, indicating the input peroxidase successfully catalysed the TMB oxidation in the presence of H_2_O_2_ produced by the isolates (Fig. 2d). However, the colours of the other three *L. fermentum* isolates did not change (Fig. 2e). Next, to examine if the inhibition of *S. mutans* growth is mediated by H_2_O_2_, the overnight-cultured H_2_O_2_-producing *L. fermentum* isolates were supplemented with 0.167 μg/μL of catalase and 3 μL of each mixture were dropped onto BHI agar on which *S. mutans* was seeded. After incubation in microaerobic conditions (5% oxygen) at 37 °C for 2 days, ZOIs disappeared and *S. mutans* formed colonies within the *L. fermentum* spot areas with equivalent colony size and density (Fig. 2f). In addition, the size of ZOI was inversely proportional to the concentration of catalase, indicating that H_2_O_2_ is mediating the inhibition of the growth of *S. mutans* (Fig. 2g).

### Inhibition of *S. mutans*-mediated artificial plaque formation

**Figure 3:**
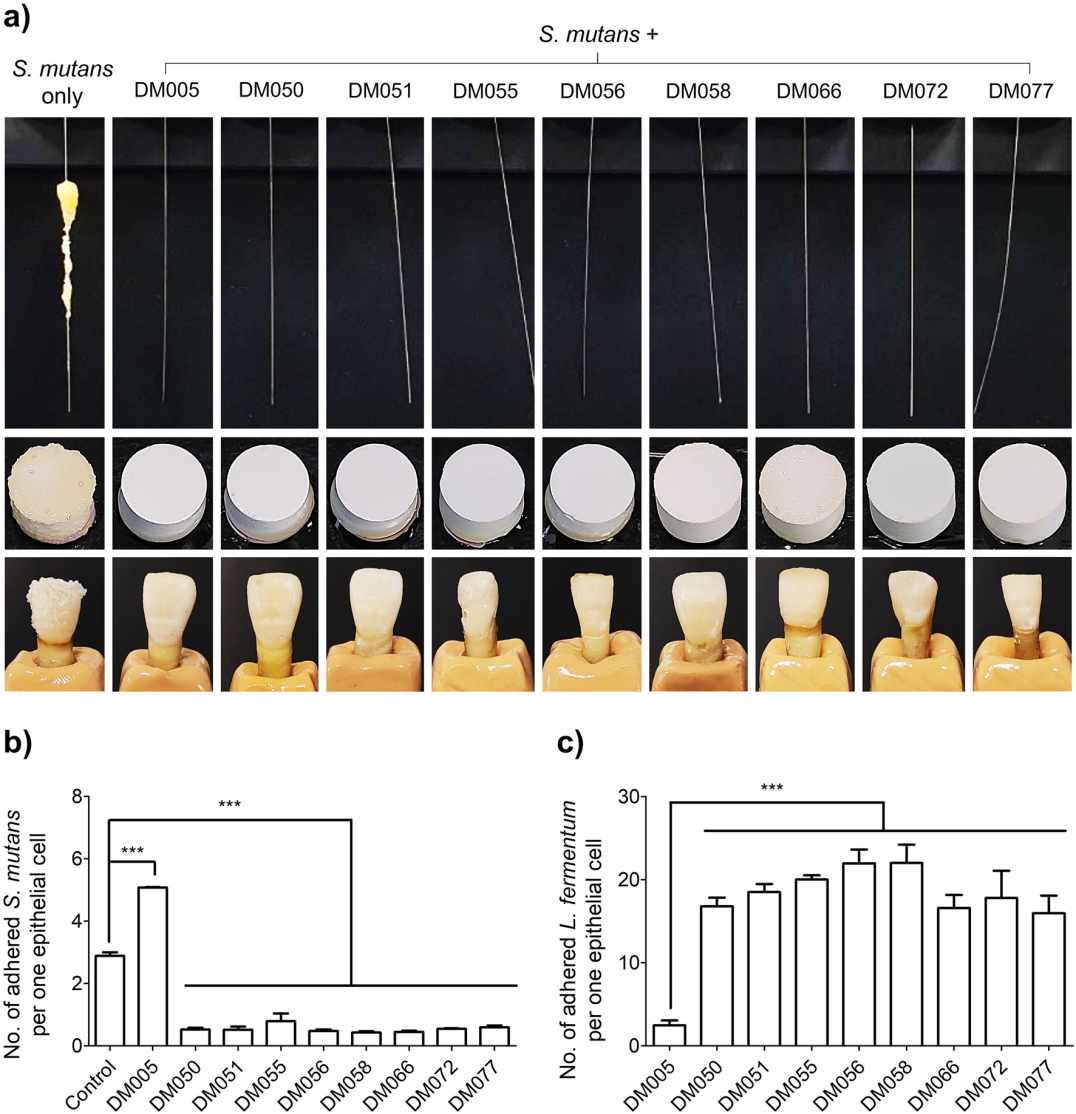
*L. fermentum* isolates inhibit *S. mutans* adherence to hard and soft surfaces. (**a**) *S. mutans* formed artificial plaque on a stainless-steel orthodontic archwire (top left), a hydroxyapatite disk (middle left), and a human tooth (bottom left) upon shake-incubation in liquid media. Co-incubation of *S. mutans* and each of the nine *L. fermentum* isolates completely suppressed the formation of artificial plaque on the wires (top), hydroxyapatite disks (middle), and human teeth (bottom). The dental numbers of the teeth used in this experiment are 11 (DM005 and DM050), 12 (DM072), 21 (*S. mutans* only, DM051, DM058, DM066), 22 (DM055), and 32 (DM056 and DM077), according to the FDI Dental Numbering System. (**b**) The *L. fermentum* isolates, except DM005, effectively kept *S. mutans* from adhering to oral epithelial KB cells. (**c**) The *L. fermentum* isolates, except DM005, adhered significantly to oral epithelial KB cells. Data were analysed using one-way ANOVA using Tukey’s multiple comparison test (***, *p* < 0.001).

To prove the inhibitory activity of the *L. fermentum* isolates against plaque formation, *S. mutans* was shake-incubated with a stainless-steel orthodontic archwire, a hydroxyapatite disk, and a human tooth in the absence or presence of each *L. fermentum* isolate. We observed a heavy biofilm formation on the wire, the hydroxyapatite disk, and the tooth when incubated with *S. mutans* only (Fig. 3a, left lanes). On the contrary, in the presence of the nine *L. fermentum* isolates (DM005 ~ DM077), the formation of biofilms on the wires, disks, and teeth was completely suppressed (Fig. 3a).

### Inhibition of *S. mutans* adherence to oral epithelial cells

Next, we tested whether *S. mutans* adherence to oral epithelial KB cells was also inhibited by the nine *L. fermentum* isolates. To that end, KB cells were co-incubated with a 1:1 mixture of *S. mutans* and each *L. fermentum* isolate and the levels of *S. mutans* attached to KB cells were assessed by quantitative PCR analysis as a ratio of colony forming unit (CFU) to one KB cell (CFU/cell). The number of *S. mutans* adhered to KB cells in the absence of *L. fermentum* isolates was 2.89 ± 0.11 CFU/cell (Fig. 3b). In contrast, when KB cells were co-incubated with *L. fermentum* isolates, the adherence of *S. mutans* to KB cells decreased (< 1.0 CFU/cell) except in the case of DM005 (5.08 ± 0.02 CFU/cell). Next, to verify if the inhibition of the *S. mutans* adherence to KB cells stems from the competitive binding of *L. fermentum* to KB cells, we incubated *L. fermentum* with KB cells and determined the number of attached *L. fermentum* cells per KB cell. As expected, *L. fermentum* DM005 adhered to KB cells at the lowest level (2.46 ± 0.33 CFU/cell) compared to the other isolates (Fig. 3c), explaining the previous results of the lowest inhibitory function of DM005 as shown in Fig. 3b. Together, these results indicated that *L. fermentum* isolates, except DM005, could adhere to the oral epithelial cells to competitively suppress *S. mutans* adherence to the oral epithelial cells. We thus excluded DM005 in the following steps for the safety evaluation.

### Safety evaluation

**Figure 4:**
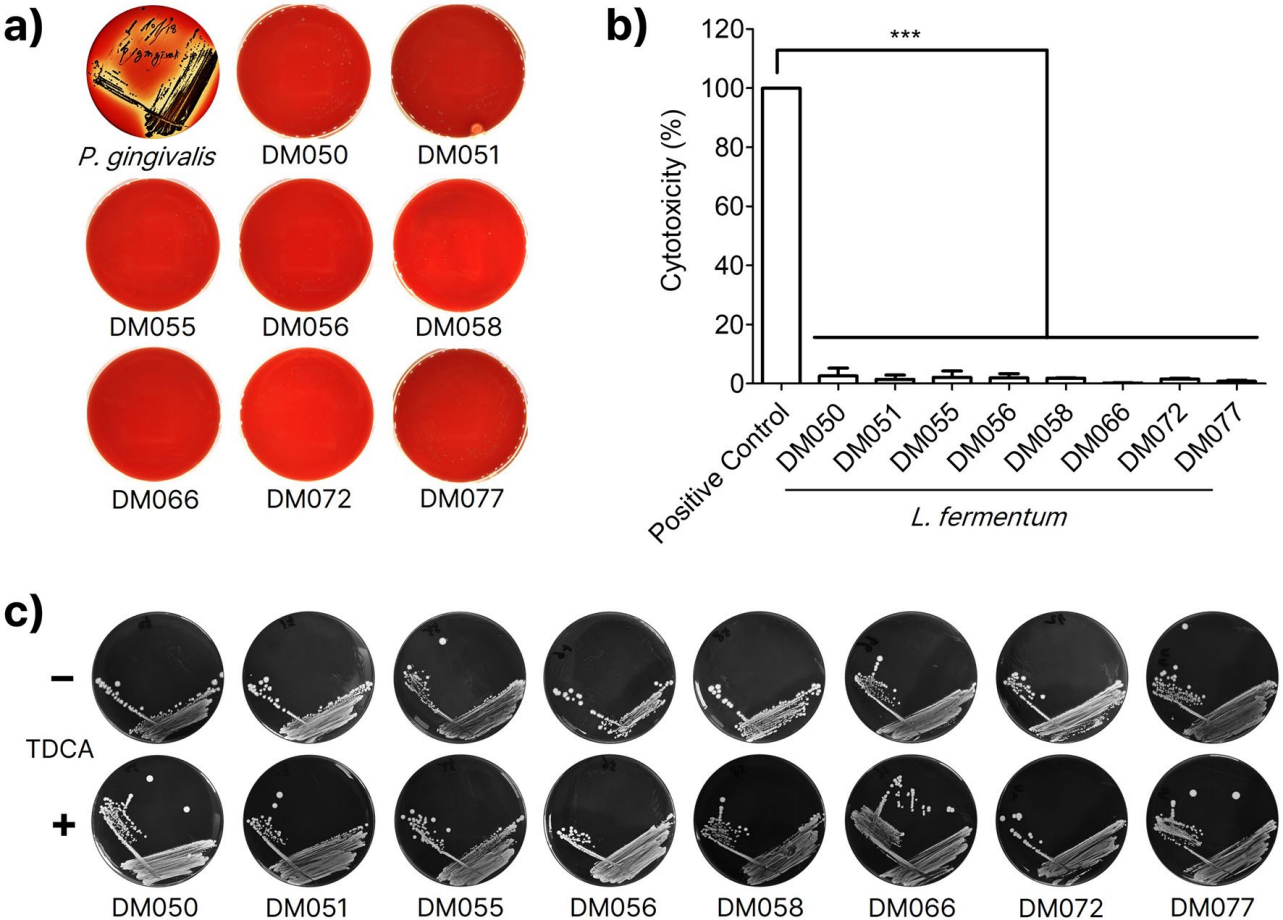
The safety evaluation of *L. fermentum* isolates. (**a**) The eight isolates showed no haemolytic activities when spread on blood agar while haemolytic *P. gingivalis* lysed blood cells to yield colourless halos around the colonies. (**b**) The eight isolates showed marginal cytotoxic effects on mammalian cells. Data were analysed by one-way ANOVA using Tukey’s multiple comparison test (***, *p* < 0.001). (**c**) The BSH activity of the eight isolates was negligible, showing no differences in appearance between colonies grown on TDCA-free and TDCA-supplemented agar plates.

No haemolytic activities were observed when the selected *L. fermentum* strains were cultured on Tryptic Soy Agar (TSA) supplemented with sheep blood for 2 days at 37 °C (Fig. 4a) ^32^. By contrast, *Porphyromonas gingivalis,* a well-known haemolytic bacterium yielded a loss of colour around the colonies. We next determined the cytotoxicity of the eight isolates by measuring cytosolic lactate dehydrogenase (LDH) released from damaged Caco-2 cells^33^. As shown in Fig. 4b, the amount of released LDH was negligible for all the isolates, indicating the isolates were non-cytotoxic to mammalian cells. Next, we qualitatively determined the bile salt hydrolase (BSH) activity using taurodeoxycholic acid (TDCA)-supplemented MRS agar^34^. No opaque halos or precipitates around the colonies nor morphological differences between colonies grown on TDCA-free and TDCA-supplemented agar plates were found, indicating that all the *L. fermentum* candidates have negligible BSH activities (Fig. 4c). In addition, the amount of D-lactate the eight isolates generate ranged from 0.01 to 0.03 mM, much lower than 0.01–0.25 mM of normal plasma levels and > 3 mM of plasma level of D-lactic acidosis (Supplementary Fig. S2)^35^.

### Antibiotic profiling

Previously, genomic analyses of DM072 using CARD (v3.2.3) and ResFinder (v4.1) yielded no antimicrobial resistance genes^36^. In this study, we tried to confirm the antibiotic susceptibility of the eight *L. fermentum* candidates employing the cut-off levels recommended by EFSA. The susceptibility and minimal inhibitory concentrations (MICs) of all the isolates were tested on a LAB-susceptibility test medium (LSM) containing a serial dilution of antibiotic compounds, such as gentamicin (GEN), kanamycin (KAN), streptomycin (STR), tetracycline (TET), erythromycin (ERY), clindamycin (CLIN), chloramphenicol (CHL), and ampicillin (AMP), and determined MICs according to the European Food Safety Authority (EFSA) criteria for obligate heterofermentative LAB. All eight strains were susceptible to all antibiotics tested in this study (Table 2). Notably, all the tested strains were the most susceptible to CLIN, a class of lincomycin, up to 32-fold low concentration (0.032 mg/L) compared to cut-off values of 1.0 mg/L (Table 2 and Supplementary Table S1). The antibiotics that showed the closest to the cut-off values were KAN (16.0 mg/L), ERY (0.5 mg/L), and CHL (2.0 mg/L), all of which were on the border of the MIC criteria. Overall, the strain DM072 was most susceptible to antibiotics tested, especially for the aminoglycoside class and tetracycline.

**Table 2:** Antibiotics resistance test of *L. fermentum* strains.

Class	Aminoglycoside			Tetracycline	Macrolide	Lincomycin	Amphenicol	β-Lactam
Antibiotic (mg/L)	GEN	KAN	STR	TET	ERY	CLIN	CHL	AMP
Cut-off value*	16	32	64	8	1	1	4	2
DM050	2	32	8	4	1	0.032	4	0.25
DM051	2	32	8	4	1	0.032	4	0.25
DM055	2	32	32	4	1	0.032	4	0.25
DM056	8	32	32	4	1	0.032	4	0.25
DM058	8	32	8	4	1	0.032	4	0.25
DM066	4	32	16	4	1	0.032	4	0.25
DM072	2	16	4	2	1	0.032	4	0.25
DM077	8	32	16	4	1	0.032	4	0.5

*Based on EFSA criteria for obligate heterofermentative *Lactobacillus* including *L. fermentum*.

### Phylogenomic and functional analyses

**Figure 5:**
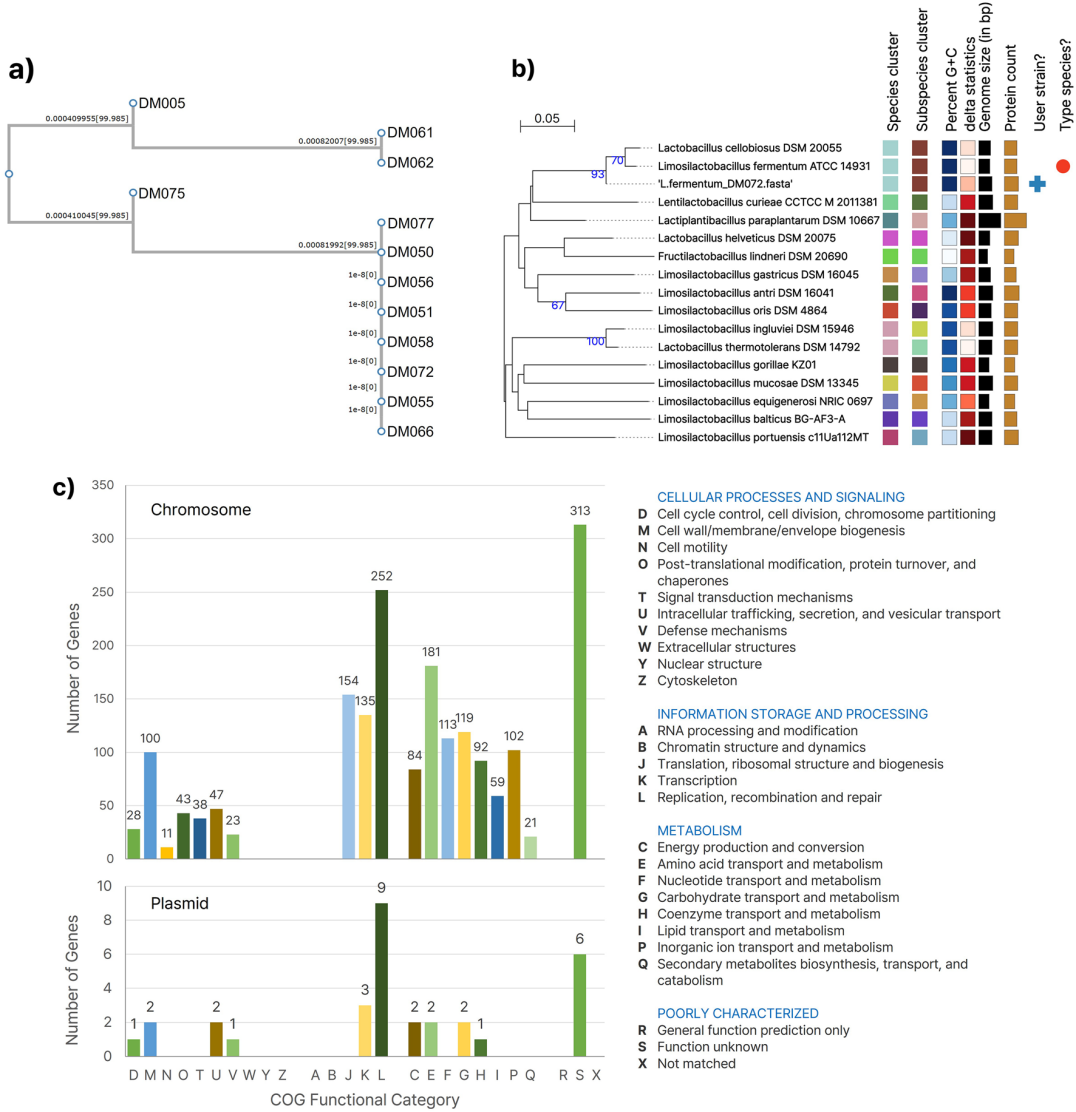
Phylogenomic analysis of *L. fermentum* isolates. (**a**) Phylogenetic relationship of 12 isolates based on 16S rRNA sequence. The midpoint-rooted tree showed that H_2_O_2_-producing DM005 diverges from the other H_2_O_2_-producing strains while having a closer homology to DM061 and DM062. The strain DM075 was also displaced from the eight H_2_O_2_-producing species clades. (**b**) The midpoint-rooted phylogenomic tree of the *L. fermentum* DM072 (GenBank accession numbers: CP102714.1 and CP102715) based on the GBDP phylogenetic analyses retrieved from the TYGS website. The numbers above branches are GBDP pseudo-bootstrap support values > 60% from 100 replications, with an average branch support of 34.9%. (c) A functional category analysis of *L. fermentum* DM072, both chromosome and plasmid, based on eggNOG/COG pipeline.

The 16S rRNA sequences of the 12 isolates and whole-genome sequence (WGS) of DM072 and DM075 (Supplementary Fig. S3) were deposited in GenBank (Supplementary Table S2). Phylogenetic analysis of the 12 isolates based on 16S rRNA sequences showed that the eight H_2_O_2_-producing strains except DM005 were grouped into one species clade (Fig. 5a). Rather, the strain DM005 was phylogenetically closer to DM061 and DM062 even if it was classified as the H_2_O_2_-producing strains. This is consistent with the inhibitory profile of the nine isolates against *S. mutans* adhesion to KB cells, where DM005 was the inactive lone strain (See Fig. 3b,c).

Previously, we calculated the average nucleotide identity (ANI) of DM072, identifying *L. fermentum* IMDO130101 as the closest strain with 99.47% sequence identity^36^. In this study, we performed WGS-based phylogenomic analyses of DM072 based on the Genome BLAST Distance Phylogeny approach (GBDP) as implemented on the Type (Strain) Genome Server (TYGS) website (Fig. 5b) ^37^. Unlike ANI analysis using OrthoANI^36^, the putative type species *L. fermentum* ATCC 14931 was placed next to DM072 strain, probably because of the differences in algorithms and databases between those tools. The functional annotation of DM072 genes was performed using eggNOG-mapper v2 based on eggNOG (v5.0)^38,39^. A total of 1790 COG-categorised genes were returned, showing 290 and 6 genes involved in cellular processes and signalling, 541 and 12 information storage and processing, 771 and 7 metabolism, and 313 and 6 poorly characterized for chromosome and plasmid, respectively (Fig. 5c).

## Discussion

Oral probiotics offer an indirect but holistic method to restore microbial ecology, thereby eliciting the healthy status of the oral cavity while minimizing unwanted side effects^40^. Conversely, the healthy oral cavity provides a higher probability of finding beneficial strains adapted to the target site where the growth of pathogens is sufficiently suppressed, as evidenced by several oral probiotic LAB isolated from the oral cavity^15,41–43^. In the case of dental caries, some LAB found in the oral cavity reduce the acid burden caused by caries-associated bacteria, such as *S. mutans,* by re-establishing pH homeostasis^44^ while others produce H_2_O_2_ at concentrations sufficient to be toxic to microorganisms lacking H_2_O_2_-scavenging enzymes^20,45,46^. In this study, we aimed to isolate LAB that have inhibitory activities against *S. mutans* from TC biospecimens of healthy volunteers. Among the isolated LAB strains, nine *L. fermentum* isolates (DM005, DM050, DM051, DM055, DM056, DM058, DM066, DM072, and DM077) suppressed the growth of *S. mutans* by producing H_2_O_2_. Intriguingly, the rest three *L. fermentum* isolates (DM061, DM062, and DM075) did not produce H_2_O_2_ and thus failed to inhibit the growth of *S. mutans*, confirming that the effective materials produced by probiotics are strain-specific^47^.

There have been various attempts to isolate LAB from the oral cavity. For example, 30 lactobacillus strains from 100 Iranian saliva samples^48^, 140 H_2_O_2_-producing LAB were isolated from the saliva of 460 healthy children in S. Korea^49^, 8 isolates from the saliva swabs from 26 healthy volunteers of Caucasian and Asian ethnicities, 500 colonies including 50 lactobacilli from the saliva of 200 adults in S. Korea^20,24^, and 896 strains of LAB from the dental plaque and tongue coatings of 32 healthy volunteers in Japan^50^. In this study, we isolated 85 LAB from 5% of the 100 human tongue coating specimens stored in VMG2 media optimised for biosample transport^51^. The apparent detection rates vary owing to the differences in sample types, the methods for sample collection, storage, and selection, as well as the purpose of the studies.

Many bacterial species in the human body produce H_2_O_2_ to serve as opportunistic pathobionts, such as *S. pyogenes*, *S. mutans*, *S. pneumoniae*^52–54^, or as beneficial commensals, such as *Bifidobacterium bifidum*, *L. johnsonii*, *L. crispatus*, *L. jensenii*, and *L. gasseri*^55–58^. The production of H_2_O_2_ occurs in energy metabolism by oxidases, such as lactate oxidase (Lox)^54^, NADH oxidase (Nox)^52,59^, pyruvate oxidase (Pox)^53^, and NADH-dependent flavin reductase^60^. Given that *S. mutans* also produces H_2_O_2_ via NADH oxidase^52^, the amount of H_2_O_2_ it produces should be regulated to stand oxidative stress^61^. By contrast, the amount of H_2_O_2_ the nine *L. fermentum* isolates produce appeared to be higher enough to suppress *S. mutans*, implying that the pathogen has low or minimal activities of H_2_O_2_-scavenging enzymes^62^. Further, a whole-genome sequencing analysis of strain DM072 revealed a gene encoding Pox^36^, implying that DM072 produce H_2_O_2_ via a Pox-dependent pathway. Intriguingly, a genomic analysis of DM075 yielded no Pox-encoding genes^63^, partially explaining the difference in H_2_O_2_ production ability between the isolated *L. fermentum* strains. However, additional genomic and transcriptomic analyses should be conducted to determine the activities of oxidases and scavengers related to H_2_O_2_ production by the nine isolates.

The initial stages of cariogenic biofilm formation on hard and soft tissues include the synthesis of adhesive glucan from sucrose by glucosyltransferases of *S. mutans* to promote the clustering of other microorganisms containing glucan-binding proteins^11^. LAB are known to inhibit pathogen growth, adhesion, and co-aggregation by the secretion of antimicrobial substances as well as the generation of an unfavourable environment for pathogens^64^. Previously, the formation of artificial plaque by *S. mutans* was partially blocked by *E. faecium* T7^65^ and fully suppressed by *L. gasseri* HHuMIN D^20^ and *L. fermentum* OK^24^. We further evaluated the inhibitory function of the isolates not only on hard tissues such as orthodontic appliances^66^, hydroxyapatite disks^67^, and extracted human teeth, but also on soft tissues such as oral epithelial cells^20,24,41,68^. As expected, co-incubation of the isolates competitively and completely prevented *S. mutans* from artificial plaque formation on all kinds of hard surfaces and adherence to KB cells (see Fig. 3). Notably, the isolates did not form a biofilm on hard surfaces while competitively adhered to soft tissues. Given that acidogenic LAB can also cause dental caries, it would be preferable for the isolates to competitively adhere to epithelial cells rather than form a biofilm on hard surfaces, allowing them to remain in the oral cavity for longer. Of note, DM005 failed to inhibit *S. mutans* attachment to KB cells, which can be explained by its low ability to adhere to KB cells (see Fig. 3b,c). Interestingly, a phylogenetic analysis of the 12 isolates using 16S rRNA sequences revealed the least likelihood of DM005 among the nine H_2_O_2_-producing strains, supporting the experimental difference between DM005 and others.

The BSH activity has often been included among the criteria for probiotic strain selection. The transformation of primary bile acids (BAs) to secondary BAs by microbial BSH plays an important role in shaping host health, greatly expanding the downstream host metabolism of lipids and glucose while possibly increasing the toxicity of bile acids^69,70^. Consequently, contradictory reports have been made regarding BSH activity owing to the widespread distribution of BSH among LAB coupled with multiple health benefits including a reduction in inflammation and blood cholesterol levels, urinary tract infections, symptoms of Crohn’s disease and atopic dermatitis^71,72^. BSH also catalyses further biotransformation of the secondary BAs such as TDCA, enabling qualitative assay of BSH activity as recommended by the Korean Ministry of Food and Drug Safety (MFDS). However, BSHs from different bacteria have their own substrate specificities, activities, and resistance to the primary and secondary BAs^70^. In our study, the selected *L. fermentum* isolates showed no precipitate originating from deconjugated TDCA in plate assay, indicating the isolates have a negligible BSH activity specific for TDCA. Also, *bsh*-related genes encoding BSH were not found in the DM072 genome when analysed by NCBI Prokaryotic Genome Annotation Pipeline (PGAP)^73^ and eggNOG (v5.0)^38^ (data not shown). It should be noted, however, that *bsh* genes can be acquired horizontally^74^ and thus the beneficial and harmful effects of bacteria that can metabolite BAs should be determined in a collective and concerted manner within their ecological niche.

Many LAB can produce L-, D-, racemate DL-lactate, or a combination of these, which have been consumed by humans as fermented foods and used for therapeutic purposes^75^. Previously, the level of D-lactate produced by 15 probiotic LAB including *Bifidobacterium*, *Lactobacillus*, *Leuconostoc*, and *Pediococcus* have been reported to range from 0.31 to 33.0 mM^76^. In one study, a widely commercialized probiotic *L. rhamnosus* GG (ATCC 53103, LGG) was reported to produce up to 7.6 mM of D-lactate in 24-h MRS culture supernatant^77^. In humans, elevated D-lactate plasma levels are responsible for rare metabolic symptomology that has only been reported in patients with short bowel syndrome^75^. However, associations between D-lactic acid bacteria and D-lactic acidosis are unclear as numerous clinical studies reported the therapeutic efficacy of D-lactic acid-producing bacteria without causing D-lactic acidosis^75^. Thus, care must be taken in the categorisation of D-lactic acid-producing bacteria as generally unsafe.

In this study, during the establishment of the oral LAB library from the TC biospecimens, we found three LAB species (*L. mucosae*, *L. sunkii*, and *L. nagelii*) not listed in HOMD (See Table 1). Given that the differences in food cultures of different regions and ethnic groups can yield different bacterial species adapted to the oral cavity, probiotic strains derived from the oral cavity may also be regio- and ethno-specific. An antimicrobial resistance profile of oral probiotic candidates could also be regiospecific owing to differences in the prescription regimen of antibiotics and the use of antibiotics in food materials between countries. On condition that the use of antibiotics should be and has been regulated, discovering probiotic strains from the oral cavity is more beneficiary because the orally adapted strains originate mostly from the edible ingredients in staple and preferred diets regardless of regionality and ethnicity. Indeed, we successfully isolated multiple *L. fermentum* candidates in the oral LAB library obtained from Korean volunteers that effectively inhibited the growth of *S. mutans* while satisfying the safety criteria, especially for antibiotics, based on EFSA guidelines. Thus, with successful in vivo and clinical studies and stability evaluations, the selected oral *L. fermentum* strains could serve as oral probiotic agents that can reverse the dysbiotic and cariogenic microbial ecology in the oral cavity while providing general probiotic benefits.

## Methods

### Isolation and identification of TC-originating LAB strains

All procedures were conducted in accordance with relevant guidelines and regulations approved by the ethics committee of the Institutional Review Board of the Apple Tree Dental Hospital (approval number: ATDH-2021–0001) and the Korea National Institute for Bioethics Policy (approval number: P01-202111-31-002). The TC biospecimens, collected from 100 healthy adults (15 males, 85 females; mean age 28.23 years; range 21–51 years) with little or no supragingival plaque, were distributed by the Biobank of Apple Tree Dental Hospital, a part of the Korea Biobank Network (KBN). The TC samples were resuspended in 4 mL of VMG2 media^51^ and stored at -80 °C until used. To isolate TC-derived LAB, 0.2 mL of TC samples in VMG2 media (5%) were diluted tenfold with Lactobacillus Selective (LBS) broth (Kisan Bio, Korea) and plated onto 20 plates of LBS agar (100 μL per plate) and anaerobically incubated for 48 h at 37 °C. The colonized isolates were identified by 16S rRNA partial sequencing (Macrogen, Inc., Korea).

### Growth inhibition of tooth-decaying pathogen by LAB isolates

*S. mutans* strain KCOM 1054 was obtained from the Korean Collection for Oral Microbiology (KCOM) of the Korea National Research Resource Centre (KNRRC). After cultivation in BD Difco™ Brain Heart Infusion (BHI) broth (Fisher Scientific, USA) under 5% microaerobic conditions (5% oxygen) at 37 °C for 24 h, the *S. mutans* cells were spread on BHI agar (Fisher Scientific, USA) to form one million colony-forming units (CFU). On top of the *S. mutans* colonies dropped 3 μL of each culture medium of the isolates, followed by incubation under microaerobic conditions (5% oxygen) at 37 °C for 24 h. The diameter of the zones of inhibition was then measured with ZEN software (Zeiss, Germany).

### Hydrogen peroxide (H_2_O_2_) production assay

H_2_O_2_ production was analysed as described by Eschenbach et al^78^ with minor modifications. In brief, selective agar plates were prepared by supplementing 1.0 mM of TMB (Sigma-Aldrich, USA) and 10 μg/mL of peroxidase (Sigma-Aldrich, USA) with MRS agar (Fisher Scientific, USA). The LAB isolates were individually diluted in MRS broth, spread on TMB-MRS selective agar plates, and incubated under anaerobic conditions at 37 °C for 48 h. The TMB-MRS plates were then exposed to air for 30 min to allow the generation of H_2_O_2_ by the selected LAB isolates, which will be consumed by peroxidase that catalyses the oxidation of TMB to yield blue colour. To confirm the effect of H_2_O_2_, 0.5 μg of catalase (Sigma-Aldrich, USA) was supplemented per 3 μL of each LAB culture medium during *S. mutans* inhibition assay. The dose-dependent effects of the catalase were tested using the lower amount of 0.25 and 0.125 μg.

### Inhibition of artificial plaque formation

Human teeth (FDI dental numbers: 11, 12, 21, 22, 32) were distributed by the Biobank of Apple Tree Dental Hospital in accordance with relevant guidelines and regulations approved by the ethics committee of the Korea National Institute for Bioethics Policy (approval number: P01-202302-02-003). Solid Stainless Steel orthodontic wires (0.016″ diameter, 14″ length) (Ultimate Wireforms, USA), hydroxyapatite discs (0.38″ diameter, 0.2″ thickness) (Clarkson Chromatography Products, PA, USA), and human teeth were autoclaved before use. The formation of artificial plaque and inhibition assay using orthodontic wires was conducted as previously described by Yu et al^65^. with minor modifications. Briefly, an equal amount (4 mL) of overnight-cultured *S. mutans* and each isolate were inoculated in a 50 mL polycarbonate tube containing 30 mL of MRS:BHI (1:1) broth supplemented with 0.1 M 4-morpholinepropanesulfonic acid (MOPS, pH 7.0) (Kisan Bio, Korea) and 5% sucrose (Sigma-Aldrich, USA). The orthodontic wires were immersed in each culture tube containing bacterial cells and shake-incubated at 100 rpm at 37 °C for 12 h. The hydroxyapatite discs were placed at the center of each well of 12-well plate containing 3 mL of culture medium and shake-incubated at 100 rpm at 37 °C for 48 h^67^. The extracted, biobanked, and distributed human teeth were fixed on 3 M™ ESPE™ Soft Putty (3 M India, India) and placed at the bottom of each 10 mL cylindrical tube containing 5 mL of culture medium and shake-incubated at 100 rpm at 37 °C for 48 h.

### Inhibition of *S. mutans* adhesion to oral epithelial cells by LAB isolates

The competitive adhesion test was performed using a modified version of a previously reported method^79^. Human oral epithelial KB cells (Korean Cell Line Bank, No.10017) were grown in RPMI1640 medium (Corning, USA) containing L-glutamine, 10% fetal bovine serum (FBS) (Atlas Biologicals, USA), and 1% antibiotic–antimycotic solution (Corning, USA). KB cells were seeded at a concentration of 2.0 × 10^5^ cells in a 6-well tissue culture plate and incubated in 5% CO_2_ at 37 °C. After 2 days at late post-confluence, the cells were washed twice with phosphate-buffered saline (PBS) and added with 2 mL of RPMI1640 containing 2% bovine serum albumin (BSA). After incubation for 2 h, the medium was changed to 1 mL of RPMI1640. KB cells from two wells were collected and their concentration was determined under optical microscopy using a haemocytometer (Marienfeld, Germany). Overnight-cultured *S. mutans* and the isolates were pelleted and resuspended with RPMI1640 at a concentration of approximately 1.0 × 10^9^ cells/mL. An equal volume (0.5 mL) of *S. mutans* and each isolate were mixed respectively and incubated under 5% CO_2_ at 37 °C. After 1 h, each well was washed three times with PBS, and 0.2 mL of trypsin/EDTA was applied to collect the KB cells and the *S. mutans* cells attached to KB cells. The genomic DNA (gDNA) was extracted from the collected cells and used for a quantitative PCR (qPCR) analysis using Exicycler™ 96 Real-Time PCR systems (Bioneer, Korea), SYBR Green PCR master mix (Thermo Fisher Scientific, USA), and primers for *S. mutans* glucosyltransferase I gene (5′-CTACACTTTCGGGTGGCTTG-3′ and 5′-GAAGCTTTTCACCATTAGAAGCTG-3′)^20^. The reaction conditions comprise the first denaturation step for 3 min at 95 °C followed by 40 cycles of a denaturation step for 10 s at 95 °C and an annealing/extension step for 20 s at 60 °C. The standard curve was created by plotting the CFU numbers of *S. mutans* against the respective mean cycle threshold (C_T_) values. Each competitive adhesion test was conducted in triplicate and the results were shown as the CFU number of *S. mutans* attached to one KB cell.

### LAB adhesion to oral epithelial cells

The adhesion test was conducted as described by Scaletsky et al^80^ with minor modifications. KB cells were grown in RPMI1640 medium containing L-glutamine, 10% FBS, and 1% antibiotic–antimycotic solution. KB cells were seeded at a concentration of 5.0 × 10^4^ cells in a 24-well tissue culture plate and incubated under 5% CO_2_ at 37 °C. After 2 days at late post-confluence, the monolayer was washed twice with PBS, and 0.5 mL of RPMI1640 containing 2% BSA was added to each well. After incubation for 2 h, the medium was changed to 0.5 mL of RPMI1640. KB cells from three wells were collected and their concentration was determined under optical microscopy using a hemocytometer (Marienfeld, Germany). Overnight-cultured LAB isolates were centrifugated and the supernatant was removed. Each pellet was resuspended with RPMI1640 at a concentration of approximately 1.0 × 10^8^ cells/mL, of which 0.5 mL was further incubated in 5% CO_2_ at 37 °C for 1 h. KB cells were then washed three times with PBS and lysed using 0.05% of Triton-X100 (Sigma-Aldrich, USA). Serial dilutions of the lysates were plated onto MRS agar plates and incubated in 5% CO_2_ at 37 °C for 24 h. The CFU number was counted to evaluate the extent to which LAB isolates adhered to KB cells. Each test was conducted in triplicate and the results were shown as the CFU number of the bacterial cells attached to one KB cell.

### Haemolysis test

Haemolysis was observed by anaerobic incubation of the isolates in TSA (Fisher Scientific, USA) supplemented with 5% sheep blood (Kisan Bio, Korea) at 37 °C for 2 days^32^. The loss of blood colour around colonies indicates haemolysis.

### Cytotoxicity test

Caco-2 cells were grown in DMEM (Corning, USA) medium containing L-glutamine, 10% FBS, and 1% antibiotic–antimycotic solution^33^. Caco-2 cells were seeded at a concentration of 1.0 × 10^4^ cells in a 96-well tissue culture plate and incubated in 5% CO_2_ at 37 °C. After 5 days at late post-confluence, Caco-2 cells were treated with overnight-cultured LAB isolates at a concentration of approximately 1.0 × 10^8^ cells and incubated in 5% CO_2_ at 37 °C. After 24 h incubation, the culture medium was centrifuged for 5 min at 3000 rpm and the supernatants were analysed for LDH measurement using LDH Activity Colorimetric Assay Kit (BioVision, USA)^81^. The lysate extracted from Caco-2 cells at late post-confluence using 0.1% Triton-X100 (Sigma-Aldrich, USA) was used as the positive control. The culture medium of Caco-2 cells at late post-confluence without LAB treatment was used as the negative control. Cytotoxicity (%) was calculated by (test sample – negative control)/(positive control – negative control) × 100.

### Bile salt hydrolase (BSH) assay

The overnight cultures of LAB strains were streaked onto MRS agar plates supplemented with 0.5% (w/v) taurodeoxycholic acid (TDCA) (Sigma-Aldrich, USA) and incubated at 37 °C for 48 h. The strains producing precipitate halos or white opaque colonies were considered BSH-active strains according to Dashkevicz et al^34^.

### Antibiotic susceptibility test

Minimal inhibitory concentration (MIC) values for LAB isolates were determined in LSM medium (90% of Iso-Sensitest broth (Kisan Bio, Korea) and 10% MRS broth) according to the ISO 10932:2010 broth microdilution procedure^82^. LSM medium was supplemented with serial dilutions of antibiotic compounds including GEN (0.5–256 mg/L), KAN (2–1024 mg/L), STR (0.5–256 mg/L), TET (2–1024 mg/L), ERY (0.016–8 mg/L), CLIN (0.032–16 mg/L), CHL (0.125–64 mg/L), and AMP (0.032–16 mg/L). Overnight-cultured LAB isolates were added at a concentration of 10^5^ CFU and anaerobically incubated at 37 °C for 2 days. MIC was determined as the lowest concentration of antimicrobial compounds at which the growth of the isolates was inhibited. The growth was measured at 600 nm absorbance using a SpectraMax iD3 microplate reader (Molecular Devices, USA) and MICs were compared to the cut-off values recommended by the EFSA^29^.

### Phylogenomic and functional analysis

Initial alignment of 16S rRNA sequences of 12 isolates was conducted using CLUSTALW (https://genome.jp/tools-bin/clustalw) and phylogenetic reconstructions were performed using the function "build" of ETE3 3.1.2^83^ implemented on the GenomeNet (https://www.genome.jp/tools/ete/). Maximum-likelihood (ML) tree was inferred using PhyML v20160115 ran with model and parameters: –pinv e –alpha e –nclasses 4 -o tlr -f m –bootstrap -2^84^. Branch supports are the Chi^2^-based parametric values returned by the approximate likelihood ratio test.

For WGS-based phylogenomic identification and phylogenomic tree construction, the WGS data of DM072 were submitted to the TYGS, a free bioinformatics platform available under https://tygs.dsmz.de, and the type-based species clustering was done using a 70% dDDH threshold^85^. The analysis also made use of recently introduced methodological updates and features^37^. The phylogenetic tree was inferred with FastME 2.1.6.1^86^ from GBDP distances calculated from genome sequences. The branch lengths are scaled in terms of the GBDP distance formula *d*_5_. Information on nomenclature, synonymy and associated taxonomic literature was provided by TYGS's sister database, the List of Prokaryotic names with Standing in Nomenclature (LPSN, available at https://lpsn.dsmz.de)^37^. The results were provided by the TYGS on 2022–12-05.

An automated functional annotation of genes was performed using eggNOG-mapper v2 based on fast orthology assignments using precomputed eggNOG 5.0 clusters and phylogenies^38,39^. The Clusters of Orthologous Groups (COG) category was extracted from eggNOG results and sorted according to the NCBI’s 25 functional categories designated from A to Z. If necessary, the NCBI PGAP^73^ was used to compare the annotation results. Circular genome maps were generated using Proksee (https://proksee.ca/).

### Statistical analysis

Data are expressed as mean ± SEM by Prism software (GraphPad Software, USA). Differences between the means were measured by one-way ANOVA using Tukey’s Multiple Comparison test. Statistical significance was assumed at *p* < 0.05.

### Ethics statement

The collection and distribution of biospecimens by the Biobank of Apple Tree Dental Hospital was approved by the ethics committee of the Apple Tree Medical Foundation (IRB number: ATDH-2021–0001). All participants understood the purpose of the study and provided informed consent. The study using the distributed TC biospecimens (IRB approval number: P01-202111-31-002) and human teeth (IRB approval number: P01-202303-02-003) were approved by the ethics committee of the Public Institutional Review Board (http://public.irb.or.kr) run by the Korea National Institute for Bioethics Policy.

## Supplementary Information

Supplementary Information.

## Data Availability

The 16S rRNA partial sequences of 12 *L. fermentum* and WGS of DM072 and DM075 are available in GenBank at the National Centre for Biotechnology Information (NCBI). The accession numbers for the 16S rRNA partial sequences of DM005, DM050, DM051, DM055, DM056, DM058, DM066, DM072, and DM077 are OP782688, OP787483, OP795824, OP795825, OP795823, OP795827, OP787485, OP579180, OP787484, OP787866, OP795828, and OP579185, respectively. The accession numbers are summarised in Supplementary Table S2.
