# Supplementary material for: Antimicrobial activity of Limosilactobacillus fermentum strains isolated from the human oral cavity against Streptococcus mutans
Source: Sci Rep. 2023 May 17;13:7969. doi: 10.1038/s41598-023-35168-7 (PMC10192434; doi:10.1038/s41598-023-35168-7)
Supplement: Supplementary file 1 — Supplementary Information. [file 41598_2023_35168_MOESM1_ESM.pdf]

Supplementary Information

Antimicrobial activity of *Limosilactobacillus fermentum* strains isolated from the human oral cavity against *Streptococcus mutans*

Do-Young Park,[a] Jiyoung Hwang,[a] Yunji Kim,[b] Dahye Lee,[c] Young-Youn Kim,[b,c] Hye-Sung Kim,[b,c] Inseong Hwang[a*]

[a] DOCSmedi Co., Ltd., Goyang-si, South Korea
[b] Apple Tree Institute of Biomedical Science, Apple Tree Medical Foundation, Goyang-si, South Korea
[c] Apple Tree Dental Hospital, Apple Tree Medical Foundation, Goyang-si, South Korea
[*] Address correspondence to Inseong Hwang, his@docsmedi.kr

Methods for Supplementary Figures S1 and S2

Acid tolerance test

The overnight cultures of LAB strains were washed twice with PBS and inoculated at a concentration of 10^8 CFU/mL into PBS adjusted to pH 3.0 and pH 2.5 with HCl. After incubation for 3 h, LAB strains were plated on MRS agar plates and incubated at 37°C for 48 h. The survival rate (%) of the strains was calculated by (CFU/mL on acidic PBS)/(CFU/mL on control PBS) × 100.

D-lactate assay

The overnight cultures of LAB strains were centrifuged at 3,000×*g* for 5 min at 4 °C and the supernatant was mixed with the agents in a D-lactate colourimetric assay kit (BioVision Inc., USA). Absorbances of the mixtures were measured at 450 nm and the concentration of D-lactate was calculated according to the manufacturer's protocol.

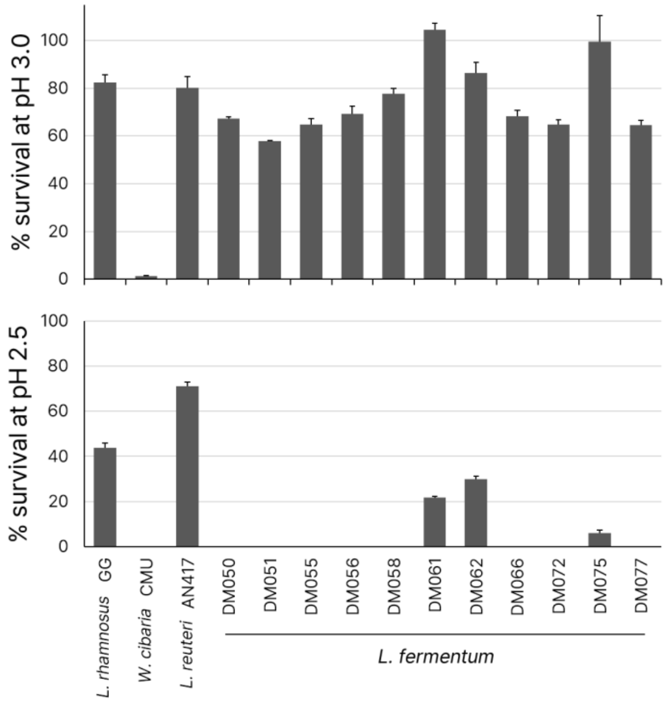

Supplementary Figure S1

Acid tolerance of LAB strains. The eight *L. fermentum* isolates were compared to commercialized *L. rhamnosus* GG (ATCC 53103) and proprietary *Weissella cibaria* CMU[1] isolated from infant saliva in South Korea and *L. reuteri* AN417 isolated from porcine intestine in South Korea[2].

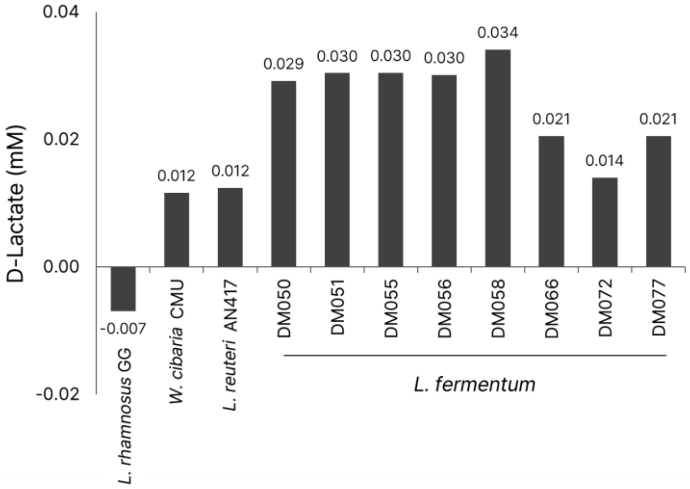

Supplementary Figure S2

Quantitative analysis of D-lactate produced by LAB strains. The eight *L. fermentum* isolates were compared to commercialized *L. rhamnosus* GG (ATCC 53103) and proprietary *Weissella cibaria* CMU[1] isolated from infant saliva in South Korea and *L. reuteri* AN417 isolated from porcine intestine in South Korea[2].

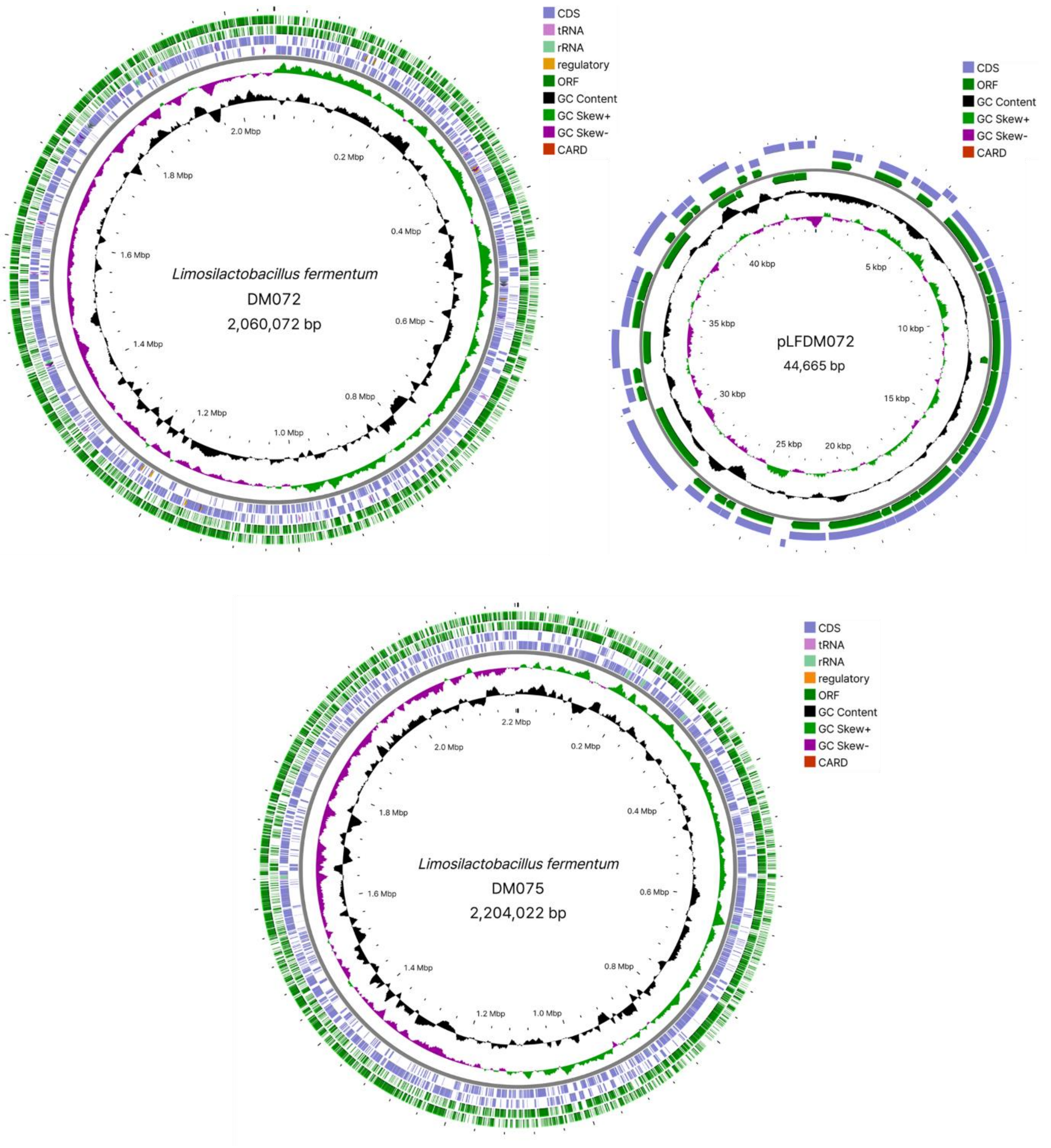

Supplementary Figure S3

Genome atlas of DM072 (GenBank accession number: CP102714.1 and CD10275) and DM075 (Genbank accession number: CP100352). The images were generated using Proksee (http://proksee.ca).

Supplementary Table S1

The cut-off values of antibiotics in LSM media (μg/mL) and OD_{600} of tested strains. The vertical thick lines indicate cut-off values for each antibiotic.

GEN (μg/mL)	0	0.5	1	2	4	8	16	32	64	128	256
DM050	0.1185	0.1337	0.1353	0.0392	0.0390	0.0389	0.0393	0.0391	0.0399	0.0402	0.0407
DM051	0.1351	0.1504	0.1374	0.0407	0.0401	0.0400	0.0401	0.0401	0.0410	0.0411	0.0408
DM055	0.1597	0.1650	0.0835	0.0416	0.0408	0.0412	0.0412	0.0410	0.0419	0.0415	0.0406
DM056	0.1344	0.1173	0.1435	0.1459	0.1380	0.0397	0.0404	0.0402	0.0394	0.0413	0.0405
DM058	0.1186	0.2584	0.2186	0.2527	0.1337	0.0403	0.0406	0.0408	0.0407	0.0414	0.0405
DM066	0.1258	0.1936	0.2328	0.3846	0.0400	0.0400	0.0403	0.0401	0.0407	0.0414	0.0403
DM072	0.1003	0.1032	0.0737	0.0416	0.0398	0.0400	0.0395	0.0392	0.0397	0.0407	0.0402
DM077	0.1792	0.2155	0.2342	0.2553	0.1386	0.0393	0.0399	0.0401	0.0406	0.0400	0.0398

KAN (μg/mL)	0	2	4	8	16	32	64	128	256	512	1024
DM050	0.1177	0.0776	0.1335	0.1373	0.1296	0.0386	0.0385	0.0386	0.0386	0.0400	0.0386
DM051	0.1312	0.0893	0.1266	0.0796	0.1069	0.0397	0.0394	0.0402	0.0399	0.0405	0.0408
DM055	0.1599	0.1486	0.1631	0.1505	0.1693	0.0408	0.0405	0.0410	0.0406	0.0409	0.0405
DM056	0.1343	0.1068	0.1251	0.1381	0.1314	0.0739	0.0398	0.0406	0.0407	0.0403	0.0394
DM058	0.0728	0.0935	0.1619	0.3104	0.2156	0.0402	0.0403	0.0409	0.0407	0.0412	0.0404
DM066	0.1283	0.0865	0.2580	0.2002	0.1767	0.0428	0.0398	0.0409	0.0401	0.0407	0.0412
DM072	0.1141	0.0622	0.1178	0.1172	0.0406	0.0398	0.0395	0.0395	0.0387	0.0400	0.0397
DM077	0.1160	0.1042	0.2404	0.2085	0.3244	0.0422	0.0398	0.0406	0.0400	0.0396	0.0401

STR (μg/mL)	0	0.5	1	2	4	8	16	32	64	128	256
DM050	0.1093	0.1000	0.0934	0.1246	0.1378	0.0385	0.0389	0.0390	0.0388	0.0385	0.0388
DM051	0.1172	0.1110	0.0772	0.1229	0.1283	0.0410	0.0401	0.0398	0.0407	0.0405	0.0400
DM055	0.1539	0.1606	0.1319	0.1720	0.1548	0.0712	0.1146	0.0405	0.0409	0.0409	0.0407
DM056	0.1211	0.1020	0.1128	0.1602	0.1012	0.1297	0.1345	0.0392	0.0398	0.0405	0.0402
DM058	0.2055	0.1419	0.1076	0.4346	0.5980	0.0490	0.0401	0.0403	0.0407	0.0415	0.0406
DM066	0.1372	0.2320	0.1001	0.2698	0.2824	0.2409	0.0402	0.0408	0.0414	0.0410	0.0408
DM072	0.1176	0.1205	0.1222	0.1061	0.0392	0.0392	0.0421	0.0411	0.0401	0.0403	0.0403
DM077	0.1161	0.1209	0.0999	0.2626	0.4000	0.3699	0.0400	0.0401	0.0405	0.0432	0.0404

TET (μg/mL)	0	0.125	0.25	0.5	1	2	4	8	16	32	64
DM050	0.1164	0.0910	0.0891	0.0837	0.0696	0.0557	0.0435	0.0438	0.0414	0.0405	0.0447
DM051	0.1169	0.1063	0.0973	0.0839	0.0782	0.0549	0.0443	0.0425	0.0434	0.0438	0.0449
DM055	0.1642	0.1347	0.1388	0.1313	0.1102	0.0755	0.0520	0.0474	0.0452	0.0448	0.0450
DM056	0.1241	0.0957	0.1098	0.1022	0.0887	0.0670	0.0492	0.0440	0.0438	0.0428	0.0434
DM058	0.2164	0.1174	0.1006	0.0929	0.0779	0.0680	0.0464	0.0436	0.0451	0.0457	0.0452
DM066	0.1390	0.1119	0.1235	0.0932	0.0800	0.0642	0.0450	0.0437	0.0442	0.0447	0.0458
DM072	0.1144	0.0634	0.0586	0.0588	0.0535	0.0497	0.0469	0.0457	0.0442	0.0422	0.0499
DM077	0.1517	0.1137	0.0987	0.1063	0.0868	0.0626	0.0456	0.0430	0.0428	0.0425	0.0438

(Continued)

ERY (µg/mL)	0	0.016	0.032	0.063	0.125	0.25	0.5	1	2	4	8
DM050	0.1123	0.0956	0.1007	0.1053	0.1079	0.1035	0.1083	0.0398	0.0400	0.0411	0.0405
DM051	0.1132	0.1111	0.1114	0.0981	0.0955	0.0982	0.0988	0.0450	0.0410	0.0420	0.0390
DM055	0.1274	0.1285	0.1410	0.1639	0.1322	0.1228	0.1522	0.0451	0.0425	0.0413	0.0428
DM056	0.1239	0.1106	0.1074	0.1162	0.1276	0.1221	0.1309	0.0462	0.0452	0.0412	0.0432
DM058	0.1180	0.1423	0.1361	0.1301	0.1354	0.1231	0.1227	0.0513	0.0453	0.0485	0.0394
DM066	0.1008	0.1344	0.1256	0.1310	0.1306	0.1425	0.1270	0.0442	0.0412	0.0464	0.0411
DM072	0.1119	0.0743	0.1121	0.0631	0.1065	0.1144	0.1034	0.0642	0.0520	0.0603	0.0618
DM077	0.1080	0.1265	0.1228	0.1232	0.1263	0.1214	0.1220	0.0544	0.0532	0.0411	0.0423

CLIN (µg/mL)	0	0.032	0.063	0.125	0.25	0.5	1	2	4	8	16
DM050	0.1119	0.0386	0.0384	0.0382	0.0384	0.0393	0.0388	0.0393	0.0397	0.0388	0.0393
DM051	0.1142	0.0405	0.0401	0.0397	0.0403	0.0395	0.0406	0.0401	0.0404	0.0411	0.0407
DM055	0.1312	0.0410	0.0410	0.0409	0.0407	0.0409	0.0411	0.0411	0.0415	0.0409	0.0414
DM056	0.1209	0.0396	0.0395	0.0398	0.0397	0.0397	0.0398	0.0403	0.0408	0.0413	0.0412
DM058	0.1854	0.0405	0.0407	0.0413	0.0411	0.0410	0.0410	0.0408	0.0415	0.0416	0.0410
DM066	0.1291	0.0403	0.0408	0.0403	0.0412	0.0417	0.0407	0.0408	0.0414	0.0410	0.0419
DM072	0.1137	0.0406	0.0394	0.0405	0.0392	0.0393	0.0396	0.0396	0.0406	0.0417	0.0422
DM077	0.1206	0.0412	0.0402	0.0403	0.0402	0.0396	0.0401	0.0403	0.0405	0.0403	0.0406

CHL (µg/mL)	0	0.125	0.25	0.5	1	2	4	8	16	32	64
DM050	0.1113	0.0955	0.0979	0.1034	0.0907	0.0867	0.0453	0.0417	0.0400	0.0377	0.0428
DM051	0.1145	0.1280	0.1127	0.0985	0.0900	0.0864	0.0426	0.0453	0.0407	0.0403	0.0399
DM055	0.1368	0.1298	0.1336	0.1265	0.1238	0.1147	0.0766	0.0447	0.0416	0.0408	0.0420
DM056	0.1216	0.1170	0.1208	0.1295	0.1209	0.1024	0.0541	0.0455	0.0417	0.0414	0.0409
DM058	0.2189	0.1311	0.1195	0.1154	0.0959	0.1121	0.0607	0.0424	0.0414	0.0408	0.0402
DM066	0.1303	0.1389	0.1206	0.1118	0.1348	0.1163	0.0639	0.0453	0.0415	0.0410	0.0407
DM072	0.1163	0.1181	0.0589	0.0557	0.1051	0.1004	0.0603	0.0410	0.0404	0.0398	0.0431
DM077	0.1211	0.1198	0.1166	0.1121	0.1116	0.1178	0.0683	0.0459	0.0418	0.0412	0.0412

AMP (µg/mL)	0	0.032	0.063	0.125	0.25	0.5	1	2	4	8	16
DM050	0.1191	0.0734	0.0586	0.0472	0.0394	0.0396	0.0400	0.0404	0.0392	0.0381	0.0397
DM051	0.1190	0.0846	0.1044	0.0497	0.0405	0.0405	0.0422	0.0412	0.0408	0.0408	0.0402
DM055	0.1359	0.0802	0.0674	0.0474	0.0405	0.1297	0.0412	0.0418	0.0417	0.0417	0.0404
DM056	0.1408	0.1216	0.1105	0.0485	0.0403	0.0388	0.0389	0.0402	0.0409	0.0406	0.0425
DM058	0.1354	0.0996	0.0612	0.0506	0.0407	0.0404	0.0402	0.0418	0.0413	0.0416	0.0408
DM066	0.1281	0.1143	0.1015	0.0491	0.0412	0.0485	0.0407	0.0405	0.0418	0.0411	0.0418
DM072	0.1088	0.1029	0.0569	0.0465	0.0398	0.0397	0.0400	0.0400	0.0403	0.0406	0.0400
DM077	0.1211	0.0832	0.1140	0.0506	0.0802	0.0407	0.0403	0.0412	0.0404	0.0398	0.0408

Supplementary Table S2

GenBank accession numbers of genes and genomes of *L. fermentum* isolates. The 16 rRNA Sanger sequencing was done using primers 27F and 1492R.

Isolated *L. fermentum*	GenBank accession numbers		
	16S rRNA	Whole-genome	
		Chromosome	Plasmid
DM005	OP782688		
DM050	OP787483		
DM051	OP795824		
DM055	OP795825		
DM056	OP795823		
DM058	OP795827		
DM066	OP787485		
DM072	OP579180	CP102714	CP102715
DM077	OP787484		
DM061	OP787866		
DM062	OP795828		
DM075	OP579185	CP100352	

1. Kang, M.-S. et al. Complete Genome Sequences of *Weissella cibaria* Strains CMU, CMS1, CMS2, and CMS3 Isolated from Infant Saliva in South Korea. *Genome Announc*. 5:e01103-17. https://doi.org/10.1128/genomeA.01103-17.
2. Yang, K. M. *et al. Lactobacillus reuteri* AN417 cell-free culture supernatant as a novel antibacterial agent targeting oral pathogenic bacteria. *Sci. Rep.* **11**, 1631 (2021). https://doi.org:10.1038/s41598-020-80921-x
